# Supplementary material for: Prevalence and Effect of Low Skeletal Muscle Mass among Hepatocellular Carcinoma Patients Undergoing Systemic Therapy: A Systematic Review and Meta-Analysis
Source: Cancers (Basel). 2023 Apr 23;15(9):2426. doi: 10.3390/cancers15092426 (PMC10177136; doi:10.3390/cancers15092426)

Prevalence and Effect of Low Skeletal-Muscle Mass among Hepatocellular Carcinoma Patients Undergoing Systemic Therapy: A Systematic Review and Meta-Analysis

Supplemental Materials

Table S1 - PRISMA checklist

Table S2 - Search strategy

Table S3 - Excluded studies and reasons

Table S4- Quality of the included cohort studies as assessed by Newcastle–Ottawa Scale.

Table S5 - Subgroup analysis of the prevalence of LSMM among HCC patients undergoing systemic therapy

Table S6 - Subgroup analysis of HR and 95% confidence interval of overall survival among HCC patients undergoing systemic therapy with and without LSMM

Table S7. Subgroup analysis HR and 95% confidence interval of progression free survival among HCC patients undergoing systemic therapy with and without LSMM

Figure S1. Publication bias

Figure S2. Sensitivity analysis

Table S1. PRISMA checklist

Section/topic	#	Checklist item	Reported on page #
**TITLE**			
Title	1	Identify the report as a systematic review, meta-analysis, or both.	Page 1
**ABSTRACT**			
Structured summary	2	Provide a structured summary including, as applicable: background; objectives; data sources; study eligibility criteria, participants, and interventions; study appraisal and synthesis methods; results; limitations; conclusions and implications of key findings; systematic review registration number.	Page 3–4
**INTRODUCTION**			
Rationale	3	Describe the rationale for the review in the context of what is already known.	Page 5
Objectives	4	Provide an explicit statement of questions being addressed with reference to participants, interventions, comparisons, outcomes, and study design (PICOS).	Page 5
**METHODS**			
Protocol and registration	5	Indicate if a review protocol exists, if and where it can be accessed (e.g., Web address), and, if available, provide registration information including registration number.	Page 3
Eligibility criteria	6	Specify study characteristics (e.g., PICOS, length of follow-up) and report characteristics (e.g., years considered, language, publication status) used as criteria for eligibility, giving rationale.	Page 6
Information sources	7	Describe all information sources (e.g., databases with dates of coverage, contact with study authors to identify additional studies) in the search and date last searched.	Page 6
Search	8	Present full electronic search strategy for at least one database, including any limits used, such that it could be repeated.	Page 6

Study selection	9	State the process for selecting studies (i.e., screening, eligibility, included in systematic review, and, if applicable, included in the meta-analysis).	Page 6-7
Data collection process	10	Describe method of data extraction from reports (e.g., piloted forms, independently, in duplicate) and any processes for obtaining and confirming data from investigators.	Page 7
Data items	11	List and define all variables for which data were sought (e.g., PICOS, funding sources) and any assumptions and simplifications made.	Page 7
Risk of bias in individual studies	12	Describe methods used for assessing risk of bias of individual studies (including specification of whether this was done at the study or outcome level), and how this information is to be used in any data synthesis.	Page 7
Summary measures	13	State the principal summary measures (e.g., risk ratio, difference in means).	Page 7
Synthesis of results	14	Describe the methods of handling data and combining results of studies, if done, including measures of consistency (e.g., I^2) for each meta-analysis.	Page 7-8
Risk of bias across studies	15	Specify any assessment of risk of bias that may affect the cumulative evidence (e.g., publication bias, selective reporting within studies).	Page 8
Additional analyses	16	Describe methods of additional analyses (e.g., sensitivity or subgroup analyses, meta-regression), if done, indicating which were pre-specified.	Page 8
**RESULTS**			
Study selection	17	Give numbers of studies screened, assessed for eligibility, and included in the review, with reasons for exclusions at each stage, ideally with a flow diagram.	Figure 1
Study characteristics	18	For each study, present characteristics for which data were extracted (e.g., study size, PICOS, follow-up period) and provide the citations.	Table 1
Risk of bias within studies	19	Present data on risk of bias of each study and, if available, any outcome level assessment (see item 12).	Table 2

Results of individual studies	20	For all outcomes considered (benefits or harms), present, for each study: (a) simple summary data for each intervention group (b) effect estimates and confidence intervals, ideally with a forest plot.	Table 1
Synthesis of results	21	Present results of each meta-analysis done, including confidence intervals and measures of consistency.	Figure 2-4<br>Appendix Table 4<br>Appendix Table 5
Risk of bias across studies	22	Present results of any assessment of risk of bias across studies (see Item 15).	Table 2
Additional analysis	23	Give results of additional analyses, if done (e.g., sensitivity or subgroup analyses, meta-regression [see Item 16]).	Appendix Figure 1<br>Appendix Figure 2
**DISCUSSION**			
Summary of evidence	24	Summarize the main findings including the strength of evidence for each main outcome; consider their relevance to key groups (e.g., healthcare providers, users, and policy makers).	Page 11
Limitations	25	Discuss limitations at study and outcome level (e.g., risk of bias), and at review-level (e.g., incomplete retrieval of identified research, reporting bias).	Page 13
Conclusions	26	Provide a general interpretation of the results in the context of other evidence, and implications for future research.	Page 14
**FUNDING**			
Funding	27	Describe sources of funding for the systematic review and other support (e.g., supply of data); role of funders for the systematic review.	Page 2

Table S2. Search strategy

Database	Keyword	Date	Results
PubMed	(liver cancer) AND ((((sarcopenia) OR (((muscle mass) AND (hand strength)) AND (walking speed))) OR ((muscle mass) AND (hand strength))) OR ((muscle mass) AND (walking speed)))	April 05,2023	411
Embase	((((("muscle mass") AND (hand strength)) AND (walking speed)) OR (("muscle mass") AND (hand strength))) OR (("muscle mass") AND (walking speed))) OR (sarcopenia)) AND (liver cancer)	April 05,2023	814
CENTRAL	(liver cancer) AND ((((sarcopenia) OR (((muscle mass) AND (hand strength)) AND (walking speed))) OR ((muscle mass) AND (hand strength))) OR ((muscle mass) AND (walking speed)))	April 05,2023	19

Table S3. Excluded studies and reasons for exclusion

Reason:

1. Hepatic tumors other than HCC
2. Not related to sarcopenia or muscle mass
3. Receiving other therapy than recommend systemic therapy
4. Absence of statistical data (hazard ratios and 95% confidence intervals) regarding the influence of pretreatment sarcopenia on OS or PFS
5. Overlapping population

Citation	Reasons
Zhuang CL, Zhang FM, Li W, Wang KH, Xu HX, Song CH, Guo ZQ, Shi HP. Associations of low handgrip strength with cancer mortality: a multicentre observational study. J Cachexia Sarcopenia Muscle. 2020 Dec;11(6):1476-1486. doi: 10.1002/jcsm.12614. Epub 2020 Sep 10. PMID: 32910535; PMCID: PMC7749566.	1
Dello SA, Lodewick TM, van Dam RM, Reisinger KW, van den Broek MA, von Meyenfeldt MF, Bemelmans MH, Olde Damink SW, Dejong CH. Sarcopenia negatively affects preoperative total functional liver volume in patients undergoing liver resection. HPB (Oxford). 2013 Mar;15(3):165-9. doi: 10.1111/j.1477-2574.2012.00517.x. Epub 2012 Jun 21. PMID: 23020663; PMCID: PMC3572275.	1
Antonelli G, Gigante E, Iavarone M, et al. Sarcopenia predicts survival in patients with advanced hepatocellular carcinoma treated with Sorafenib. Journal of Hepatology 2018;68:S207-S208.	1
D'Arcangelo F, Zanetto A, Aliberti C, et al. IMPACT OF PRE-TRANSPLANT SARCOPENIA ON SHORT TO MEDIUM TERM OUTCOME IN LIVER TRANSPLANT RECIPIENTS WITH AND WITHOUT HEPATOCELLULAR CARCINOMA. Digestive and Liver Disease 2020;52:S159.	1
Kamo N, Kaido T, Hamaguchi Y, et al. Impact of sarcopenic obesity on outcomes in patients undergoing living donor liver transplantation. Clinical Nutrition 2019;38:2202-2209.	1
Valero V, Amini N, Spolverato G, et al. Sarcopenia adversely impacts postoperative complications following resection or transplantation in patients with primary liver tumors. Journal of gastrointestinal surgery : official journal of the Society for Surgery of the Alimentary Tract 2015;19:272-281.	1
Xiao LS, Li RN, Cui H, et al. Use of computed tomography-derived body composition to determine the prognosis of patients with primary liver cancer treated with immune checkpoint inhibitors: a retrospective cohort study. BMC Cancer 2022;22	1

Dodson R, Firoozmand A, Hyder O, et al. Impact of sarcopenia on outcomes following intra-arterial therapy of hepatic malignancies. HPB 2013;15:18.	1
Ha Y, Chon YE, Lee YB, et al. Sarcopenia as a predictor of survival and an objective measure of performance status in hepatocellular carcinoma. Liver Cancer 2016;5:65.	1
Heil J, Heid F, Bechstein WO, et al. Sarcopenia predicts reduced liver growth and reduced resectability in patients undergoing portal vein embolization before liver resection - A DRAGON collaborative analysis of 306 patients. HPB (Oxford) 2022;24:413-421.	1
Montano-Loza A, Meza-Junco J, Prado C, et al. Frequency and clinical impact of sarcopenia in cirrhotic patients with and without hepatocellular carcinoma. American Journal of Gastroenterology 2011;106:S125-S126.	1
Montano-Loza A, Meza-Junco J, Tandon P, et al. Muscle wasting is not associated with higher mortality after liver transplantation. American Journal of Gastroenterology 2012;107:S162.	1
Meza-Junco J, Montano-Loza A, Prado C, et al. Sarcopenia in cirrhotic patients with and without hepatocellular carcinoma. Journal of Cachexia, Sarcopenia and Muscle 2011;2:218.	1
Clement DSVM, Leerdam MEV, de Jong S, Weickert MO, Ramage JK, Tesselaar MET, Srirajaskanthan R. Prevalence of Sarcopenia and Impact on Survival in Patients with Metastatic Gastroenteropancreatic Neuroendocrine Tumours. Cancers (Basel). 2023 Jan 27;15(3):782. doi: 10.3390/cancers15030782. PMID: 36765740; PMCID: PMC9913815.	1
Gagnat G, Hobeika C, Modzelewski R, Collet CS, Di Fiore F, Druesne L, Tuech JJ, Schwarz L. Evaluation of sarcopenia biomarkers in older patients undergoing major surgery for digestive cancer. SAXO prospective cohort study. Eur J Surg Oncol. 2023 Jan;49(1):285-292. doi: 10.1016/j.ejso.2022.08.038. Epub 2022 Sep 6. PMID: 36167704.	1
Guarneri G, Pecorelli N, Bettinelli A, Campisi A, Palumbo D, Genova L, Gasparini G, Provinciali L, Della Corte A, Abati M, Aleotti F, Crippa S, De Cobelli F, Falconi M. Prognostic value of preoperative CT scan derived body composition measures in resected pancreatic cancer. Eur J Surg Oncol. 2023 Feb 13:S0748-7983(23)00137-3. doi: 10.1016/j.ejso.2023.02.005. Epub ahead of print. PMID: 36863915.	1
Hayano K, Ohira G, Kano M, Suito H, Matsumoto Y, Kurata Y, Otsuka R, Isozaki T, Toyozumi T, Murakami K, Uesato M, Matsubara H. Prognostic Impact of Hepatic Steatosis Evaluated by CT on Immunotherapy for Gastric Cancer: Associations with Sarcopenia, Systemic Inflammation, and Hormones. Oncology. 2023;101(3):185-192. doi: 10.1159/000528005. Epub 2022 Nov 15. PMID: 36380615.	1
Keyl J, Hosch R, Berger A, Ester O, Greiner T, Bogner S, Treckmann J, Ting S, Schumacher B, Albers D, Markus P, Wiesweg M, Forsting M, Nensa F, Schuler M, Kasper S, Kleesiek J. Deep learning-based assessment of body composition and liver tumour burden for survival modelling in	1

advanced colorectal cancer. J Cachexia Sarcopenia Muscle. 2023 Feb;14(1):545-552. doi: 10.1002/jcsm.13158. Epub 2022 Dec 21. PMID: 36544260; PMCID: PMC9891942.	
Su CH, Chen WM, Chen MC, Shia BC, Wu SY. The Impact of Sarcopenia Onset Prior to Cancer Diagnosis on Cancer Survival: A National Population-Based Cohort Study Using Propensity Score Matching. Nutrients. 2023 Mar 1;15(5):1247. doi: 10.3390/nu15051247. PMID: 36904245; PMCID: PMC10005798.	1
Tanaka K, Taoda A, Kashiwagi H. The impact of acute skeletal muscle loss after gastrointestinal cancer surgery on physical function. PM R. 2023 Feb;15(2):184-191. doi: 10.1002/pmrj.12774. Epub 2022 Feb 25. PMID: 35077013.	1
Thormann M, Heitmann F, Wrobel V, Barajas Ordonez F, Pech M, Surov A, Damm R, Omari J. Sarcopenia does not limit overall survival in patients with colorectal liver metastases undergoing interstitial brachytherapy. Rofo. 2023 Mar;195(3):217-223. English. doi: 10.1055/a-1936-2937. Epub 2022 Oct 25. Erratum in: Rofo. 2023 Mar;195(3):e1. PMID: 36283404.	1
Wang X, Zheng J, Yang H, Yang X, Cai W, Chen X, Zhang W, Shen X. Prognostic value of the preoperative albumin-bilirubin score among patients with stages I-III gastric cancer undergoing radical resection: A retrospective study. Clin Transl Sci. 2023 Feb 10. doi: 10.1111/cts.13493. Epub ahead of print. PMID: 36762709.	1
Bernardi L, Roesel R, Vagelli F, Majno-Hurst P, Cristaudi A. Imaging based body composition profiling and outcomes after oncologic liver surgery. Front Oncol. 2022 Dec 8;12:1007771. doi: 10.3389/fonc.2022.1007771. PMID: 36568174; PMCID: PMC9773835.	1
Lee EK, Kang YE, Park YJ, Koo BS, Chung KW, Ku EJ, Won HR, Yoo WS, Jeon E, Paek SH, Lee YS, Lim DM, Suh YJ, Park HK, Kim HJ, Kim BH, Kim M, Kim SW, Yi KH, Park SK, Jung EJ, Choi JY, Bae JS, Hong JH, Nam KH, Lee YK, Yu HW, Go S, Kang YM; MASTER study group. A Multicenter, Randomized, Controlled Trial for Assessing the Usefulness of Suppressing Thyroid Stimulating Hormone Target Levels after Thyroid Lobectomy in Low to Intermediate Risk Thyroid Cancer Patients (MASTER): A Study Protocol. Endocrinol Metab (Seoul). 2021 Jun;36(3):574-581. doi: 10.3803/EnM.2020.943. Epub 2021 May 26. PMID: 34034365; PMCID: PMC8258337.	1
O'Neill L, Guinan E, Doyle S, Connolly D, O'Sullivan J, Bennett A, Sheill G, Segurado R, Knapp P, Fairman C, Normand C, Geoghegan J, Conlon K, Reynolds JV, Hussey J. Rehabilitation strategies following oesophagogastric and Hepatopancreaticobiliary cancer (ReStOre II): a protocol for a randomized controlled trial. BMC Cancer. 2020 May 13;20(1):415. doi: 10.1186/s12885-020-06889-z. PMID: 32404096; PMCID: PMC7222585.	1
Slooter, M. D., et al. "Quantitative fluorescence-guided perfusion assessment of the gastric conduit to predict anastomotic complications after esophagectomy." Diseases of the Esophagus 34.5 (2021): doaa100.	1
Schink K, Herrmann HJ, Schwappacher R, Meyer J, Orlemann T, Waldmann E, Wullich B, Kahlmeyer A, Fietkau R, Lubgan D, Beckmann MW, Hack	1

C, Kemmler W, Siebler J, Neurath MF, Zopf Y. Effects of whole-body electromyostimulation combined with individualized nutritional support on body composition in patients with advanced cancer: a controlled pilot trial. BMC Cancer. 2018 Sep 12;18(1):886. doi: 10.1186/s12885-018-4790-y. PMID: 30208857; PMCID: PMC6134788.	
The effect of perioperative oral management on the outcomes in patients diagnosed as sarcopenia with hepato-biliary-pancreatic cancers.(https://center6.umin.ac.jp/cgi-open-bin/ctr_e/ctr_view.cgi?recptno=R000035575)	1
Rhee H, Navaratnam A, Oleinikova I, Gilroy D, Scuderi Y, Heathcote P, Nguyen T, Wood S, Ho KKY. A Novel Liver-targeted Testosterone Therapy for Sarcopenia in Androgen Deprived Men With Prostate Cancer. J Endocr Soc. 2021 Jun 19;5(9):bvab116. doi: 10.1210/jendso/bvab116. PMID: 34308090; PMCID: PMC8294688.	1
Abe S, Kawai K, Nozawa H, Sasaki K, Murono K, Emoto S, Kishikawa J, Ishii H, Yokoyama Y, Nagai Y, Anzai H, Sonoda H, Oba K, Ishihara S. Preoperative sarcopenia is a poor prognostic factor in lower rectal cancer patients undergoing neoadjuvant chemoradiotherapy: a retrospective study. Int J Clin Oncol. 2022 Jan;27(1):141-153. doi: 10.1007/s10147-021-02062-z. Epub 2021 Nov 6. PMID: 34741193.	1D
Zheng X, Cao F, Qian L, et al. Body Composition Changes in Hepatocellular Carcinoma: Prediction of Survival to Transcatheter Arterial Chemoembolization in Combination With Clinical Prognostic Factors. Cancer Control 2021;28.	2
Hashida R, Kawaguchi T, Koya S, et al. Impact of cancer rehabilitation on the prognosis of patients with hepatocellular carcinoma. Oncology Letters 2020;19:2355-2367.	2
Bettinger D, Spode R, Glaser N, et al. Survival benefit of transarterial chemoembolization in patients with metastatic hepatocellular carcinoma: A single center experience. BMC Gastroenterology 2017;17.	2
Takada H, Kurosaki M, Nakanishi H, Takahashi Y, Itakura J, Tsuchiya K, Yasui Y, Tamaki N, Takaura K, Komiyama Y, Higuchi M, Kubota Y, Wang W, Okada M, Enomoto N, Izumi N. Impact of pre-sarcopenia in sorafenib treatment for advanced hepatocellular carcinoma. PLoS One. 2018 Jun 18;13(6):e0198812. doi: 10.1371/journal.pone.0198812. PMID: 29912922; PMCID: PMC6005492.	4
Mir O, Coriat R, Blanchet B, Durand JP, Boudou-Rouquette P, Michels J, Ropert S, Vidal M, Pol S, Chaussade S, Goldwasser F. Sarcopenia predicts early dose-limiting toxicities and pharmacokinetics of sorafenib in patients with hepatocellular carcinoma. PLoS One. 2012;7(5):e37563. doi: 10.1371/journal.pone.0037563. Epub 2012 May 30. PMID: 22666367; PMCID: PMC3364283.	4
Yamashima M, Miyaaki H, Honda T, Shibata H, Miuma S, Taura N, Nakao K. Significance of psoas muscle thickness as an indicator of muscle atrophy in patients with hepatocellular carcinoma treated with sorafenib. Mol Clin Oncol. 2017 Sep;7(3):449-453. doi: 10.3892/mco.2017.1321. Epub 2017 Jul 13. PMID: 28781818; PMCID: PMC5530307.	4

Uojima H, Chuma M, Tanaka Y, Hidaka H, Nakazawa T, Iwabuchi S, Kobayashi S, Hattori N, Ogushi K, Morimoto M, Kagawa T, Tanaka K, Kako M, Koizumi W. Skeletal Muscle Mass Influences Tolerability and Prognosis in Hepatocellular Carcinoma Patients Treated with Lenvatinib. Liver Cancer. 2020 Apr;9(2):193-206. doi: 10.1159/000504604. Epub 2019 Dec 6. Erratum in: Liver Cancer. 2022 Oct 4;11(6):581. PMID: 32399433; PMCID: PMC7206580.	4
Amanuma M, Nagai H, Igarashi Y. Sorafenib Might Induce Sarcopenia in Patients With Hepatocellular Carcinoma by Inhibiting Carnitine Absorption. Anticancer Res. 2020 Jul;40(7):4173-4182. doi: 10.21873/anticanres.14417. PMID: 32620667.	4
Yamaoka K, Kodama K, Kawaoka T, Kosaka M, Johira Y, Shirane Y, Miura R, Yano S, Murakami S, Amioka K, Naruto K, Ando Y, Kosaka Y, Uchikawa S, Uchida T, Fujino H, Nakahara T, Murakami E, Okamoto W, Yamauchi M, Miki D, Imamura M, Takahashi S, Nagao A, Chayama K, Aikata H. The importance of body composition assessment for patients with advanced hepatocellular carcinoma by bioelectrical impedance analysis in lenvatinib treatment. PLoS One. 2022 Jan 18;17(1):e0262675. doi: 10.1371/journal.pone.0262675. PMID: 35041693; PMCID: PMC8765661.	4
Matsumoto H, Tsuchiya K, Hayakawa Y, et al. Clinical impact of the changes of muscle volume during various systemic therapies in patients with unresectable hepatocellular carcinoma. Journal of Clinical Oncology 2022;40.	4
Qayyum A, Bhosale P, Aslam R, Avritscher R, Ma J, Pagel MD, Sun J, Mohamed Y, Rashid A, Beretta L, Kaseb AO. Effect of sarcopenia on systemic targeted therapy response in patients with advanced hepatocellular carcinoma. Abdom Radiol (NY). 2021 Mar;46(3):1008-1015. doi: 10.1007/s00261-020-02751-9. Epub 2020 Sep 24. PMID: 32974761; PMCID: PMC8191337.	4
Ari D, Dağli M, Gökcan H, et al. Effects of pretransplant sarcopenia to clinical outcomes after liver transplantation. Turkish Journal of Gastroenterology 2019;30:S6.	4
Kobayashi A, Kaido T, Hamaguchi Y, Okumura S, Taura K, Hatano E, Okajima H, Uemoto S. Impact of postoperative changes in sarcopenic factors on outcomes after hepatectomy for hepatocellular carcinoma. J Hepatobiliary Pancreat Sci. 2016 Jan;23(1):57-64. doi: 10.1002/jhbp.302. Epub 2015 Dec 3. PMID: 26572789.	4
Imai K, Takai K, Hanai T, et al. Skeletal muscle depletion predicts the prognosis of patients with hepatocellular carcinoma treated with sorafenib. International Journal of Molecular Sciences 2015;16:9612-9624.	4
Harimoto N, Hoshino H, Muranushi R, Hagiwara K, Yamanaka T, Ishii N, Tsukagoshi M, Igarashi T, Watanabe A, Kubo N, Araki K, Shirabe K. Skeletal Muscle Volume and Intramuscular Adipose Tissue Are Prognostic Predictors of Postoperative Complications After Hepatic Resection. Anticancer	4

Res. 2018 Aug;38(8):4933-4939. doi: 10.21873/anticanres.12810. PMID: 30061272.	
Imai K, Takai K, Hanai T, Ideta T, Miyazaki T, Kochi T, Suetsugu A, Shiraki M, Shimizu M. Skeletal muscle depletion predicts the prognosis of patients with hepatocellular carcinoma treated with sorafenib. Int J Mol Sci. 2015 Apr 28;16(5):9612-24. doi: 10.3390/ijms16059612. PMID: 25927582; PMCID: PMC4463608.	5
Saeki I, Yamasaki T, Maeda M, Kawano R, Hisanaga T, Iwamoto T, Matsumoto T, Hidaka I, Ishikawa T, Takami T, Sakaida I. No Muscle Depletion with High Visceral Fat as a Novel Beneficial Biomarker of Sorafenib for Hepatocellular Carcinoma. Liver Cancer. 2018 Oct;7(4):359-371. doi: 10.1159/000487858. Epub 2018 Apr 6. PMID: 30488024; PMCID: PMC6249591.	5

citation	reason	Treatment
Badran H, Elsabaawy MM, Ragab A, et al. Baseline Sarcopenia is Associated with Lack of Response to Therapy, Liver Decompensation and High Mortality in Hepatocellular Carcinoma Patients. Asian Pac J Cancer Prev 2020;21:3285-3290.	3	All treatment
Hirota K, Kawaguchi T, Koya S, et al. Clinical utility of the Liver Frailty Index for predicting muscle atrophy in chronic liver disease patients with hepatocellular carcinoma. Hepatology Research 2020;50:330-341.	3	All treatment
Sano A, Tsuge S, Kakazu E, et al. Plasma free amino acids are associated with sarcopenia in the course of hepatocellular carcinoma recurrence. Nutrition 2021;84.	3	All treatment
Takada H, Amemiya F, Yasumura T, et al. Relationship between presarcopenia and event occurrence in patients with primary hepatocellular carcinoma. Scientific reports 2020;10:10186.	3	All treatment
Mardian Y, Yano Y, Ratnasari N, et al. "sarcopenia and intramuscular fat deposition are associated with poor survival in Indonesian patients with hepatocellular carcinoma: A retrospective study". BMC Gastroenterology 2019;19.	3	All treatment
Zhao M, Duan X, Han X, et al. Sarcopenia and Systemic Inflammation Response Index Predict Response to Systemic Therapy for Hepatocellular Carcinoma and Are Associated With Immune Cells. Frontiers in Oncology 2022;12.	3	All treatment
Imai K, Takai K, Watanabe S, et al. Sarcopenia impairs prognosis of patients with hepatocellular carcinoma: The role of liver functional reserve and tumor-related factors in loss of skeletal muscle volume. Nutrients 2017;9.	3	All treatment
Marasco G, Dajti E, Serenari M, et al. Sarcopenia Predicts Major Complications after Resection for Primary Hepatocellular Carcinoma in Compensated Cirrhosis. Cancers 2022;14.	3	All treatment

Ha Y, Kim D, Han S, et al. Sarcopenia Predicts Prognosis in Patients with Newly Diagnosed Hepatocellular Carcinoma, Independent of Tumor Stage and Liver Function. Cancer Res Treat 2018;50:843-851.	3	All treatment
Begini P, Gigante E, Antonelli G, et al. Sarcopenia predicts reduced survival in patients with hepatocellular carcinoma at first diagnosis. Ann Hepatol 2017;16:107-114.	3	All treatment
Fujiwara N, Nakagawa H, Kudo Y, et al. Sarcopenia, intramuscular fat deposition, and visceral adiposity independently predict the outcomes of hepatocellular carcinoma. Journal of Hepatology 2015;63:131-140.	3	All treatment
Iritani S, Imai K, Takai K, et al. Skeletal muscle depletion is an independent prognostic factor for hepatocellular carcinoma. Journal of Gastroenterology 2015;50:323-332.	3	All treatment
Nagamatsu A, Kawaguchi T, Hirota K, et al. Slow walking speed overlapped with low handgrip strength in chronic liver disease patients with hepatocellular carcinoma. Hepatology Research 2019;49:1427-1440.	3	All treatment
Dhooge M, Coriat R, Mir O, et al. Feasibility of gemcitabine plus oxaliplatin in advanced hepatocellular carcinoma patients with child-pugh B cirrhosis. Oncology (Switzerland) 2013;84:32-38.	3	Chemotherapy
Mir O, Coriat R, Boudou-Rouquette P, et al. Gemcitabine and oxaliplatin as second-line treatment in patients with hepatocellular carcinoma pre-treated with sorafenib. Med Oncol 2012;29:2793-9.	3	Chemotherapy
Kamachi S, Mizuta T, Otsuka T, et al. Sarcopenia is a risk factor for the recurrence of hepatocellular carcinoma after curative treatment. Hepatology Research 2016;46:201-208.	3	Curative treatment
Santhakumar C, Bartlett AS, Plank LD, et al. Sarcopenia negatively impacts long-term outcomes following curative resection for hepatocellular carcinoma: Results of a long-term follow-up study. GastroHep 2020;2:215-223.	3	Curative treatment
Kim H, Choi HZ, Choi JM, et al. Sarcopenia with systemic inflammation can predict survival in patients with hepatocellular carcinoma undergoing curative resection. Journal of Gastrointestinal Oncology 2022;13:744-753.	3	Curative treatment
Salman A, Salman M, Moustafa A, et al. Impact of sarcopenia on two-year mortality in patients with hcv-associated hepatocellular carcinoma after radiofrequency ablation. Journal of Hepatocellular Carcinoma 2021;8:313-320.	3	Curative treatment-RFA
Yeh WS, Chiang PL, Kee KM, et al. Pre-sarcopenia is the prognostic factor of overall survival in early-stage hepatoma patients undergoing radiofrequency ablation. Medicine (United States) 2020;99.	3	Curative treatment-RFA
Liao C, Li G, Bai Y, et al. Prognostic value and association of sarcopenic obesity and systemic inflammatory indexes in patients with hepatocellular carcinoma following hepatectomy and the establishment of novel predictive nomograms.	3	Curative treatment-surgery

Journal of Gastrointestinal Oncology 2021;12:669-693.		
Yabusaki N, Fujii T, Yamada S, et al. Adverse impact of low skeletal muscle index on the prognosis of hepatocellular carcinoma after hepatic resection. International Journal of Surgery 2016;30:136-142.	3	Curative treatment-surgery
Berardi G, Antonelli G, Colasanti M, et al. Association of Sarcopenia and Body Composition with Short-term Outcomes after Liver Resection for Malignant Tumors. JAMA Surgery 2020;155.	3	Curative treatment-surgery
Hayashi H, Shimizu A, Kubota K, et al. Combination of sarcopenia and prognostic nutritional index to predict long-term outcomes in patients undergoing initial hepatectomy for hepatocellular carcinoma. Asian journal of surgery 2022.	3	Curative treatment-surgery
Seror M, Sartoris R, Hobeika C, et al. Computed Tomography-Derived Liver Surface Nodularity and Sarcopenia as Prognostic Factors in Patients with Resectable Metabolic Syndrome-Related Hepatocellular Carcinoma. Annals of Surgical Oncology 2021;28:405-416	3	Curative treatment-surgery
Itoh S, Shirabe K, Matsumoto Y, et al. Effect of body composition on outcomes after hepatic resection for hepatocellular carcinoma. Ann Surg Oncol 2014;21:3063-8.	3	Curative treatment-surgery
Kroh A, Uschner D, Lodewick T, et al. Impact of body composition on survival and morbidity after liver resection in hepatocellular carcinoma patients. Hepatobiliary and Pancreatic Diseases International 2019;18:28-37.	3	Curative treatment-surgery
Bekki T, Abe T, Amano H, et al. Impact of low skeletal muscle mass index and perioperative blood transfusion on the prognosis for HCC following curative resection. BMC Gastroenterology 2020;20.	3	Curative treatment-surgery
Hiraoka A, Otsuka Y, Kawasaki H, et al. Impact of muscle volume and muscle function decline in patients undergoing surgical resection for hepatocellular carcinoma. Journal of Gastroenterology and Hepatology (Australia) 2018;33:1271-1276.	3	Curative treatment-surgery
Omiya S, Komatsu S, Kido M, et al. Impact of sarcopenia as a prognostic factor on reductive hepatectomy for advanced hepatocellular carcinoma. Anticancer Research 2021;41:5775-5783.	3	Curative treatment-surgery
Yang J, Chen K, Zheng C, et al. Impact of sarcopenia on outcomes of patients undergoing liver resection for hepatocellular carcinoma. J Cachexia Sarcopenia Muscle 2022;13:2383-2392.	3	Curative treatment-surgery
Kobayashi A, Kaido T, Hamaguchi Y, et al. Impact of Sarcopenic Obesity on Outcomes in Patients Undergoing Hepatectomy for Hepatocellular Carcinoma. Annals of Surgery 2019;269:924-931.	3	Curative treatment-surgery
Hamaguchi Y, Kaido T, Okumura S, et al. Muscle Steatosis is an Independent Predictor of Postoperative Complications	3	Curative treatment-

in Patients with Hepatocellular Carcinoma. World journal of surgery 2016;40:1959-1968.		surgery
Bajric T, Wagner D, Faschinger F, et al. Neodadjuvant therapy influences sceletal muscle mass after liver resection for malignancies. European Journal of Surgical Oncology 2022;48:e123	3	Curative treatment-surgery
Kubota R, Kumamoto T, Takeda K, et al. Pre-operative assessment of muscle mass to predict prognosis in patient with hepatocellular carcinoma. HPB 2015;17:134.	3	Curative treatment-surgery
Hamaguchi Y, Kaido T, Okumura S, et al. Preoperative intramuscular adipose tissue content is a novel prognostic predictor after hepatectomy for hepatocellular carcinoma. Clinical Nutrition 2015;34:S11.	3	Curative treatment-surgery
Hamaguchi Y, Kaido T, Okumura S, et al. Preoperative muscle steatosis is a novel prognostic predictor after hepatectomy for hepatocellular carcinoma. HPB 2016;18:e215-e216.	3	Curative treatment-surgery
Hamaguchi Y, Kaido T, Okumura S, et al. Preoperative Visceral Adiposity and Muscularity Predict Poor Outcomes after Hepatectomy for Hepatocellular Carcinoma. Liver Cancer 2019;8:92-109.	3	Curative treatment-surgery
Takagi K, Yagi T, Yoshida R, et al. Sarcopenia and American Society of Anesthesiologists Physical Status in the Assessment of Outcomes of Hepatocellular Carcinoma Patients Undergoing Hepatectomy. Acta medica Okayama 2016;70:363-370.	3	Curative treatment-surgery
Jang HY, Choi GH, Hwang SH, et al. Sarcopenia and visceral adiposity predict poor overall survival in hepatocellular carcinoma patients after curative hepatic resection. Translational Cancer Research 2021;10:854-866.	3	Curative treatment-surgery
Harimoto N, Shirabe K, Yamashita YI, et al. Sarcopenia as a predictor of prognosis in patients following hepatectomy for hepatocellular carcinoma. British Journal of Surgery 2013;100:1523-1530.	3	Curative treatment-surgery
Voron T, Tselikas L, Pietrasz D, et al. Sarcopenia impacts on short- and long-term results of hepatectomy for hepatocellular carcinoma. Annals of Surgery 2015;261:1173-1183.	3	Curative treatment-surgery
Salman MA, Omar HSE, Mikhail HMS, et al. Sarcopenia increases 1-year mortality after surgical resection of hepatocellular carcinoma. ANZ J Surg 2020;90:781-785.	3	Curative treatment-surgery
Harimoto N, Yoshizumi T, Shimokawa M, et al. Sarcopenia is a poor prognostic factor following hepatic resection in patients aged 70 years and older with hepatocellular carcinoma. Hepatology Research 2016;46:1247-1255.	3	Curative treatment-surgery
Meister FA, Lurje G, Verhoeven S, et al. The Role of Sarcopenia and Myosteatosis in Short-and Long-Term Outcomes Following Curative-Intent Surgery for Hepatocellular Carcinoma in a European Cohort. Cancers 2022;14.	3	Curative treatment-surgery

Yanagaki M, Haruki K, Taniai T, et al. The significance of osteosarcopenia as a predictor of the long-term outcomes in hepatocellular carcinoma after hepatic resection. Journal of Hepato-Biliary-Pancreatic Sciences 2022.	3	Curative treatment-surgery
Hayashi H, Shimizu A, Kubota K, Notake T, Masuo H, Yoshizawa T, Hosoda K, Sakai H, Yasukawa K, Soejima Y. Combination of sarcopenia and prognostic nutritional index to predict long-term outcomes in patients undergoing initial hepatectomy for hepatocellular carcinoma. Asian J Surg. 2023 Feb;46(2):816-823. doi: 10.1016/j.asjsur.2022.07.122. Epub 2022 Aug 10. PMID: 35961897.	3	Curative treatment-surgery
Wu DH, Liao CY, Wang DF, Huang L, Li G, Chen JZ, Wang L, Lin TS, Lai JL, Zhou SQ, Qiu FN, Zhang ZB, Chen YL, Wang YD, Zheng XC, Tian YF, Chen S. Textbook outcomes of hepatocellular carcinoma patients with sarcopenia: A multicenter analysis. Eur J Surg Oncol. 2023 Apr;49(4):802-810. doi: 10.1016/j.ejso.2022.12.009. Epub 2022 Dec 22. PMID: 36586787.	3	Curative treatment-surgery
Yang J, Wang D, Ma L, An X, Hu Z, Zhu H, Zhang W, Chen K, Ma J, Yang Y, Wu L, Chen G, Wang Y. Sarcopenia negatively affects postoperative short-term outcomes of patients with non-cirrhosis liver cancer. BMC Cancer. 2023 Mar 6;23(1):212. doi: 10.1186/s12885-023-10643-6. PMID: 36879265; PMCID: PMC9987146.	3	Curative treatment-surgery
Xing J, Wan X, Sang X, Yang H, Du S. Perioperative management of patients with liver cancer complicated with sarcopenia. Hepatobiliary Surg Nutr. 2022 Dec;11(6):906-908. doi: 10.21037/hbsn-22-485. PMID: 36523921; PMCID: PMC9745625.	3	Curative treatment-surgery
Gau RY, Tsai HI, Yu MC, Chan KM, Lee WC, Wang HE, Wang SF, Cheng ML, Chiu CC, Chen HY, Lee CW. Laparoscopic liver resection is associated with less significant muscle loss than the conventional open approach. World J Surg Oncol. 2022 Dec 4;20(1):385. doi: 10.1186/s12957-022-02854-1. PMID: 36464698; PMCID: PMC9721003.	3	Curative treatment-surgery
Feng X, Feng Q, Li J. Platelet lymphocyte ratio and sarcopenia were associated with survival after hepatocellular carcinoma undergoing curative resection. J Gastrointest Oncol. 2022 Oct;13(5):2691-2692. doi: 10.21037/jgo-22-604. PMID: 36388682; PMCID: PMC9660086.	3	Curative treatment-surgery
Levolger S, van Vledder MG, Muslem R, et al. Sarcopenia impairs survival in patients with potentially curable hepatocellular carcinoma. J Surg Oncol 2015;112:208-13.	3	Curative treatment-surgery/RFA
Acosta LF, Galuppo R, García CR, et al. Association Between Sarcopenia and AFP Level in Patients Undergoing Liver Transplantation for Hepatocellular Carcinoma. J Surg Res 2019;238:10-15.	3	Curative treatment-transplant

Itoh S, Yoshizumi T, Kimura K, et al. Effect of sarcopenic obesity on outcomes of living-donor liver transplantation for hepatocellular carcinoma. Anticancer Research 2016;36:3029-3034.	3	Curative treatment-transplant
Grąt K, Pacho R, Grąt M, et al. Impact of body composition on the risk of hepatocellular carcinoma recurrence after liver transplantation. Journal of Clinical Medicine 2019;8.	3	Curative treatment-transplant
Beumer BR, van Vugt JLA, Sapisochin G, et al. Impact of muscle mass on survival of patients with hepatocellular carcinoma after liver transplantation beyond the Milan criteria. Journal of Cachexia, Sarcopenia and Muscle 2022;13:2373-2382.	3	Curative treatment-transplant
Kaido T, Hamaguchi Y, Okumura S, et al. Impact of sarcopenia in HBP and transplant surgery. Journal of Hepato-Biliary-Pancreatic Sciences 2017;24:A40.	3	Curative treatment-transplant
Kaido T, Uemoto S. Impact of sarcopenia in liver surgery. Liver Cancer 2015;4:143.	3	Curative treatment-transplant
Alsebaey A, Sabry A, Rashed HS, et al. MELD-Sarcopenia is Better than ALBI and MELD Score in Patients with Hepatocellular Carcinoma Awaiting Liver Transplantation. Asian Pacific journal of cancer prevention : APJCP 2021;22:2005-2009.	3	Curative treatment-transplant
Toshima T, Yoshizumi T, Kosai-Fujimoto Y, et al. Prognostic Impact of Osteopenia in Patients Who Underwent Living Donor Liver Transplantation for Hepatocellular Carcinoma. World journal of surgery 2020;44:258-267.	3	Curative treatment-transplant
Kim YR, Park S, Han S, et al. Sarcopenia as a predictor of post-transplant tumor recurrence after living donor liver transplantation for hepatocellular carcinoma beyond the Milan criteria. Scientific reports 2018;8:7157.	3	Curative treatment-transplant
Meza-Junco J, Montano-Loza AJ, Baracos VE, et al. Sarcopenia as a prognostic index of nutritional status in concurrent cirrhosis and hepatocellular carcinoma. J Clin Gastroenterol 2013;47:861-70.	3	Curative treatment-transplant
Tan Y, Duan T, Li B, et al. Sarcopenia defined by psoas muscle index independently predicts long-term survival after living donor liver transplantation in male recipients. Quantitative Imaging in Medicine and Surgery 2022;12:215-228.	3	Curative treatment-transplant
Huang Y, Wang N, Xu L, Wu Y, Li H, Jiang L, Xu M. Albumin-Globulin Score Combined with Skeletal Muscle Index as a Novel Prognostic Marker for Hepatocellular Carcinoma Patients Undergoing Liver Transplantation. J Clin Med. 2023 Mar 14;12(6):2237. doi: 10.3390/jcm12062237. PMID: 36983238; PMCID: PMC10051871.	3	Curative treatment-transplant
Lin Z, Li H, He C, Yang M, Chen H, Yang X, Zhuo J, Shen W, Hu Z, Pan L, Wei X, Lu D, Zheng S, Xu X. Metabolomic	3	Curative treatment-

biomarkers for the diagnosis and post-transplant outcomes of AFP negative hepatocellular carcinoma. Front Oncol. 2023 Feb 9;13:1072775. doi: 10.3389/fonc.2023.1072775. PMID: 36845695; PMCID: PMC9947281.		transplant
Lymberopoulos P, Prakash S, Shaikh A, Bhatnagar A, Allam AK, Goli K, Goss JA, Kanwal F, Rana A, Kowdley KV, Jalal P, George Cholankeril. Long-term outcomes and trends in liver transplantation for hereditary hemochromatosis in the United States. Liver Transpl. 2023 Jan 1;29(1):15-25. doi: 10.1002/lt.26539. Epub 2022 Jul 21. PMID: 35770428; PMCID: PMC9800641.	3	Curative treatment-transplant
Sim JH, Kwon HM, Kim KW, Ko YS, Jun IG, Kim SH, Kim KS, Moon YJ, Song JG, Hwang GS. Associations of sarcopenia with graft failure and mortality in patients undergoing living donor liver transplantation. Liver Transpl. 2022 Aug;28(8):1345-1355. doi: 10.1002/lt.26447. Epub 2022 Apr 25. PMID: 35243771.	3	Curative treatment-transplant
Tomiyama T, Harada N, Toshima T, Nakayama Y, Toshida K, Morinaga A, Kosai-Fujimoto Y, Tomino T, Kurihara T, Takeishi K, Nagao Y, Morita K, Itoh S, Yoshizumi T. Donor Skeletal Muscle Quality Affects Graft Mortality After Living Donor Liver Transplantation- A Single Center, Retrospective Study. Transpl Int. 2022 Dec 9;35:10723. doi: 10.3389/ti.2022.10723. PMID: 36568139; PMCID: PMC9784912.	3	Curative treatment-transplant
Botrus G, Uson PLS, Kosiorek H, et al. Prognostic impact of Sarcopenia on clinical outcomes in advanced hepatocellular carcinoma (HCC) treated with systemic therapy. Cancer Research 2022;82.	3	immunotherapy/target therapy
Cheng TY, Lee PC, Chen YT, et al. Pre-sarcopenia determines post-progression outcomes in advanced hepatocellular carcinoma after sorafenib failure. Scientific reports 2020;10:18375.	3	Post Sorafenib
Yang S, Zhang Z, Su T, et al. CT-based skeletal muscle loss for predicting poor survival in patients with hepatocellular carcinoma experiencing curative hepatectomy plus adjuvant transarterial chemoembolization: a preliminary retrospective study. European Journal of Medical Research 2022;27.	3	Radioembolization
Li LQ, Zhao WD, Su TS, et al. Effect of Body Composition on Outcomes in Patients with Hepatocellular Carcinoma Undergoing Radiotherapy: A Retrospective Study. Nutrition and Cancer 2022;74:3302-3311.	3	Radioembolization
Loannides P, Abuodeh Y, Jin W, et al. Impact of sarcopenia evaluated using the total psoas area (TPA) in patients undergoing Y-90 radioembolization for hepatocellular carcinoma (HCC). Journal of Clinical Oncology 2016;34.	3	Radioembolization
Roth G, Teyssier Y, Benhamou M, et al. Impact of sarcopenia on tumor response and survival outcomes in patients with hepatocellular carcinoma treated by trans-arterial (chemo)-embolization. World Journal of Gastroenterology	3	Radioembolization

2022;28:5324-5337.		
Zhang JX, Yan HT, Ding Y, et al. Low Psoas-Muscle index is associated with decreased survival in hepatocellular carcinoma treated with transarterial chemoembolization. Annals of Medicine 2022;54:1562-1569.	3	Radioembolization
Guichet PL, Taslakian B, Zhan C, et al. MRI-Derived Sarcopenia Associated with Increased Mortality Following Yttrium-90 Radioembolization of Hepatocellular Carcinoma. CardioVascular and Interventional Radiology 2021;44:1561-1569.	3	Radioembolization
Shiba S, Shibuya K, Katoh H, et al. No deterioration in clinical outcomes of carbon ion radiotherapy for sarcopenia patients with hepatocellular carcinoma. Anticancer Research 2018;38:3579-3586.	3	Radioembolization
Kobayashi T, Kawai H, Nakano O, et al. Rapidly declining skeletal muscle mass predicts poor prognosis of hepatocellular carcinoma treated with transcatheter intra-arterial therapies. BMC Cancer 2018;18:756.	3	Radioembolization
Lanza E, Masetti C, Messana G, et al. Sarcopenia as a predictor of survival in patients undergoing bland transarterial embolization for unresectable hepatocellular carcinoma. PLoS ONE [Electronic Resource] 2020;15:e0232371.	3	Radioembolization
Loosen SH, Schulze-Hagen M, Bruners P, et al. Sarcopenia is a negative prognostic factor in patients undergoing transarterial chemoembolization (TACE) for hepatic malignancies. Cancers 2019;11.	3	Radioembolization
Lee J, Park S, Cho Y, et al. Skeletal muscle depletion predicts the prognosis of patients with hepatocellular carcinoma treated with radiotherapy. Liver Cancer 2018;7:198.	3	Radioembolization
Fujita M, Takahashi A, Hayashi M, et al. Skeletal muscle volume loss during transarterial chemoembolization predicts poor prognosis in patients with hepatocellular carcinoma. Hepatology Research 2019;49:778-786.	3	Radioembolization
Chien TP, Huang SF, Chan WH, et al. The combination of sarcopenia and biochemical factors can predict the survival of hepatocellular carcinoma patients receiving transarterial chemoembolization. Frontiers in Oncology 2022;12.	3	Radioembolization
Lim J, Kim KW, Ko Y, et al. The role of muscle depletion and visceral adiposity in HCC patients aged 65 and over undergoing TACE. BMC Cancer 2021;21:1164.	3	Radioembolization
Ebadi M, Moctezuma-Velazquez C, Meza-Junco J, et al. Visceral adipose tissue radiodensity is linked to mortality in hepatocellular carcinoma patients treated with selective internal radiation therapy (SIRT). Canadian Liver Journal 2019;2:114-115.	3	Radioembolization
Faron A, Sprinkart AM, Pieper CC, et al. Yttrium-90 radioembolization for hepatocellular carcinoma: Outcome prediction with MRI derived fat-free muscle area. European Journal of Radiology 2020;125.	3	Radioembolization

Araki K, Harimoto N, Shibuya K, Watanabe A, Tsukagoshi M, Ishii N, Hagiwara K, Muranushi R, Hoshino K, Seki T, Shirabe K. Significance of Skeletal Muscle Loss in Liver Hypertrophy in Patients Undergoing Portal Vein Embolization Before Major Hepatectomy: Assessment With Body Composition and Nutritional Indicators. Anticancer Res. 2023 Jan;43(1):209-216. doi: 10.21873/anticanres.16151. PMID: 36585158.	3	Radioembolization
Bannangkoon K, Hongsakul K, Tubtawee T, Ina N, Chichareon P. Association of myosteatosis with treatment response and survival in patients with hepatocellular carcinoma undergoing chemoembolization: a retrospective cohort study. Sci Rep. 2023 Mar 9;13(1):3978. doi: 10.1038/s41598-023-31184-9. PMID: 36894658; PMCID: PMC9998862.	3	Radioembolization
Huang YL, Huang MC, Chang CI, Yang LH, Wu CJ, Chiu CC, Chen CY, Hsu JS, Lee KT, Chang WT. Elevated intramuscular adipose tissue content with a high Ishak fibrosis stage (>3) had a negative effect on liver regeneration in cirrhotic patients undergoing portal vein embolization. Kaohsiung J Med Sci. 2023 Feb;39(2):182-190. doi: 10.1002/kjm2.12622. Epub 2022 Nov 17. PMID: 36394149.	3	Radioembolization
Loosen SH, Jördens MS, Schoon B, Antoch G, Luedde T, Minko P, Loberg C, Roderburg C. Sarcopenia indicate poor survival in patients undergoing transarterial chemoembolization (TACE) for hepatic malignancies. J Cancer Res Clin Oncol. 2023 Jan 23. doi: 10.1007/s00432-022-04519-8. Epub ahead of print. PMID: 36689060.	3	Radioembolization
Masetti C, Pugliese N, Lofino L, Colapietro F, Ceriani R, Lleo A, Poretti D, Pedicini V, De Nicola S, Torzilli G, Rimassa L, Aghemo A, Lanza E. Myosteatosis Is Not Associated with Complications or Survival in HCC Patients Undergoing Trans Arterial Embolization. J Clin Med. 2022 Dec 29;12(1):262. doi: 10.3390/jcm12010262. PMID: 36615062; PMCID: PMC9821378.	3	Radioembolization
Nugent K, Good J. The oligometastatic paradigm and the role of radiotherapy. Clin Med (Lond). 2023 Jan;23(1):61-64. doi: 10.7861/clinmed.2022-0559. PMID: 36697003.	3	Radioembolization
Masetti C, Pugliese N, Lofino L, Colapietro F, Ceriani R, Lleo A, Poretti D, Pedicini V, De Nicola S, Torzilli G, Rimassa L, Aghemo A, Lanza E. Myosteatosis Is Not Associated with Complications or Survival in HCC Patients Undergoing Trans Arterial Embolization. J Clin Med. 2022 Dec 29;12(1):262. doi: 10.3390/jcm12010262. PMID: 36615062; PMCID: PMC9821378.	3	Radioembolization
Yang JF, Huang WY, Lo CH, Lee MS, Lin CS, Shen PC, Dai YH, Wang YF, Chen TW. Significant muscle loss after stereotactic body radiotherapy predicts worse survival in patients with hepatocellular carcinoma. Sci Rep. 2022 Nov 9;12(1):19100.	3	Radioembolization

doi: 10.1038/s41598-022-21443-6. PMID: 36352042; PMCID: PMC9646692.		
Koya S, Kawaguchi T, Hashida R, Hirota K, Bekki M, Goto E, Yamada M, Sugimoto M, Hayashi S, Goshima N, Yoshiyama T, Otsuka T, Nozoe R, Nagamatsu A, Nakano D, Shirono T, Shimose S, Iwamoto H, Niizeki T, Matsuse H, Koga H, Miura H, Shiba N, Torimura T. Effects of in-hospital exercise on sarcopenia in hepatoma patients who underwent transcatheter arterial chemoembolization. J Gastroenterol Hepatol. 2019 Mar;34(3):580-588. doi: 10.1111/jgh.14538. Epub 2018 Dec 16. PMID: 30402913.	3	Radioembolization
Antonelli G, Gigante E, Iavarone M, et al. Sarcopenia is associated with reduced survival in patients with advanced hepatocellular carcinoma undergoing sorafenib treatment. United European Gastroenterol J 2018;6:1039-1048.	3	Sorafenib
Nault JC, Pigneur F, Nelson AC, et al. Visceral fat area predicts survival in patients with advanced hepatocellular carcinoma treated with tyrosine kinase inhibitors. Dig Liver Dis 2015;47:869-76.	3	Sorafenib
Sugama Y, Miyanishi K, Osuga T, et al. Combination of psoas muscle mass index and neutrophil/lymphocyte ratio as a prognostic predictor for patients undergoing nonsurgical hepatocellular carcinoma therapy. JGH Open 2021;5:1335-1343.	3	Target-HAIC, TACE,TKI

HAIC, hepatic arterial infusion chemotherapy; HCC, hepatocellular carcinoma; OS, overall survival; PFS, progression-free survival; TACE, transarterial chemoembolization; TKI, tyrosine kinase inhibitor

Table S4. Quality of the included cohort studies as assessed by Newcastle–Ottawa Scale.

Type of systemic therapy	First author	Selection				Comparability		Outcome			Total points	Study quality
		Q1	Q2	Q3	Q4	Q1	Q2	Q1	Q2	Q3		
Sorafenib	Hiraoka (2017)	1	1	1	1	1	1	1	1	0	8	good
	Nishikawa (2017)	1	1	1	1	1	1	1	1	1	9	good
	Naganumaa (2017)	0	1	1	1	0	1	1	1	0	6	good
	Antonelli (2018)	1	1	1	1	1	1	1	1	1	9	good
	Imai (2019)	0	1	1	1	1	1	1	1	1	8	good
	Labeur (2019)	1	1	1	1	1	1	1	1	0	8	good
	Sawada (2019)	0	1	1	1	1	1	1	1	0	7	good
	Wu (2021)	0	1	1	1	0	1	1	1	0	6	good
	Saeki (2021)	1	1	1	1	1	1	1	1	1	9	good
	Ogushi (2022)	0	1	1	1	1	1	1	1	0	7	good
Lenvatinib	Endo (2020)	0	1	1	1	1	1	1	0	1	7	good
	Hiraoka (2021)	1	1	1	1	1	1	1	1	0	8	good
	Dongs (2022)	0	1	1	1	0	1	1	0	0	5	poor
	Fujita (2022)	1	1	1	1	1	1	1	1	0	8	good
	Toshida (2022)	0	1	1	1	1	1	1	1	0	7	good
Immunotherapy	Akce (2021)	0	1	1	1	1	1	1	1	0	7	good
	Kim (2021)	0	1	1	1	1	1	1	1	1	8	good
	Matsumoto (2022)	0	1	1	1	1	1	1	0	0	6	poor
	Guo (2022)	0	1	1	1	1	1	1	0	0	6	poor
	Chen (2023)	0	1	1	1	1	1	1	1	0	7	good

Studies with 3 or 4 stars in selection domain AND 1 or 2 stars in comparability domain AND 2 or 3 stars in outcome/exposure domain were judged as good quality;

studies with 2 stars in selection domain AND 1 or 2 stars in comparability domain AND 2 or 3 stars in outcome/exposure domain were judged as moderate

quality;
studies with 0 or 1 star in selection domain OR 0 stars in comparability domain OR 0 or 1 stars in outcome/exposure domain were judged as low quality.
The questions in each domain were as follows.

Selection:
Q1. Representativeness of exposed cohort: 1, truly or somewhat representative of a population-based or multi-center study; 0, selected group of users or lack of description of the derivation of the cohort.
Q2. Selection of non-exposed cohort: 1, drawn from the same community as the exposed cohort; 0, drawn from a different source or lack of description of the derivation of the non-exposed cohort.
Q3. Ascertainment of exposure: 1, secure record or structured interview; 0, written self-report or lack of description of validation.
Q4. Demonstration that outcome of interest was not present at start of study: 1, yes; 0, no.

Comparability:
Q1. Study adjusted for age and sex: 1, yes; 0, no
Q2. study controls for any additional factor: 1, yes; 0, no.

Outcome:
Q1. Assessment of outcome: 1, independent blind assessment, confirmed by medical records or record linkage; 0, self-reported or no description.
Q2. Was follow-up long enough for outcomes to occur: 1, duration of follow-up at least 2 years; 0, duration of follow-up < 2 years.
Q3. Loss to follow-up rate: 1, complete follow-up of loss to follow-up rate less than 20%; 0, loss to follow-up rate more than 20% or no statement.

Table S5. Subgroup analysis of the prevalence of LSMM among HCC patients undergoing systemic therapy

Subgroup	Records (N)	Patients (N)	Prevalence (%)	95% CI	I^2 (%)
**Overall**	21	2377	43.4	37.0 – 50.0	89.27
**Treatment regimen**					
Sorafenib	10	1496	41.7	32.0–52.1	92.88
Lenvatinib	5	447	45.5	32.1–59.5	87.34
Immunotherapy	6	434	44.4	34.0-55.3	78.65
**Study region**					
Asian country	18	1946	42.2	34.7-50.1	90.63
Non-Asian country	3	431	51.0	46.3–55.7	0
**Muscle measured**					
SMI	17	1894	44.5	37.7-51.6	87.85
PMI	4	483	38.9	22.7–58.0	93.69
**Study quality**					
Good	18	2208	42.4	35.3–49.9	90.77
Poor	3	169	49.1	41.6-56.6	0
**Observation time**					
≥2 years	17	2145	42.9	35.5–50.6	91.16
<2 years	4	232	45.5	36.8-54.4	42.95

SMI, Skeletal muscle mass index; PMI, psoas mass index; CI, confidence interval

Table S6. Subgroup analysis of HR and 95% confidence interval of overall survival among HCC patients undergoing systemic therapy with and without LSMM

Subgroup	Records (N)	Patients (N)	HR	95% CI	p	I^2 (%)
**Overall**	20	2248	1.70	1.46–1.97	< 0.001	29.27
**Crude**	16	1730	1.68	1.44–1.95	< 0.001	24.37
**Adjusted**	16	1633	1.84	1.59–2.13	< 0.001	11.07
**Treatment regimen**						
Sorafenib	11	1496	1.74	1.41–2.14	< 0.001	48.15
Lenvatinib	5	447	1.71	1.22–2.41	0.002	15.13
Immunotherapy	4	305	1.61	1.15–2.24	0.005	< 0.01
**Study region**						
Asian country	17	1817	1.80	1.54–2.11	< 0.001	15.54
Non-Asian country	3	431	1.31	1.07–1.62	0.010	< 0.01
**Muscle measured**						
SMI	16	1,765	1.74	1.44–2.10	< 0.001	42.00
PMI	4	483	1.61	1.22–2.11	0.001	< 0.01
**Observation time**						
≥ 2 years	18	2,145	1.69	1.46–1.96	< 0.001	28.24
< 2 years	2	103	1.94	0.54–6.90	0.308	68.12

All OS studies were of "good" quality

SMI, Skeletal muscle mass index; PMI, psoas mass index; CI, confidence interval

Table S7. Subgroup analysis HR and 95% confidence interval of progression free survival among HCC patients undergoing systemic therapy with and without LSMM

Subgroup	Records (N)	Patients (N)	HR	95% CI	p	I^2 (%)
**Overall**	13	1,400	1.32	1.16-1.51	< 0.001	< 0.01
**Crude**	9	882	1.57	1.24-1.98	< 0.001	42.37
**Adjusted**	7	801	1.32	1.10-1.59	0.003	< 0.01
**Treatment regimen**						
Sorafenib	5	863	1.23	1.03–1.46	0.020	< 0.01
Lenvatinib	2	103	2.08	1.18–3.67	0.012	< 0.01
Immunotherapy	6	434	1.41	1.12-1.78	0.004	< 0.01
**Study region**						
Asian area	12	1,343	1.34	1.17-1.54	< 0.001	< 0.01
Non-Asian area	1	57	0.99	0.53–1.83	0.974	< 0.01
**Study quality**						
Good	10	1,231	1.27	1.10–1.46	0.001	< 0.01
Poor	3	169	1.87	1.24-2.81	0.003	< 0.0
**Observation time**						
Over 2 years	10	1,231	1.27	1.10–1.46	< 0.001	< 0.01
Less than 2 years	3	169	1.87	1.24-2.81	0.003	< 0.01

All PFS studies used SMI.

SMI, Skeletal muscle mass index; CI, confidence interval

Figure S1. Publication bias analysis using funnel plots for

(A) Prevalence, (B) HRs of overall survival, (C) HR of progression-free survival

(A) Prevalence (Egger's test: p,0.10)

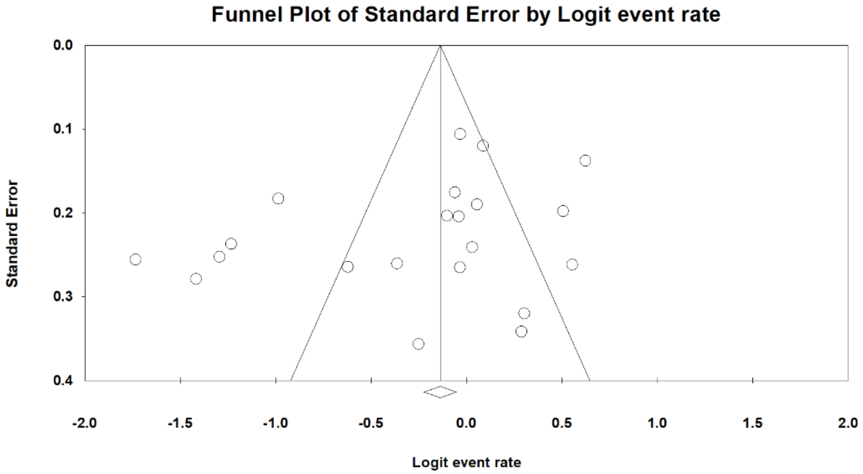

(B) HRs of overall survival (Egger's test: p, 0.20)

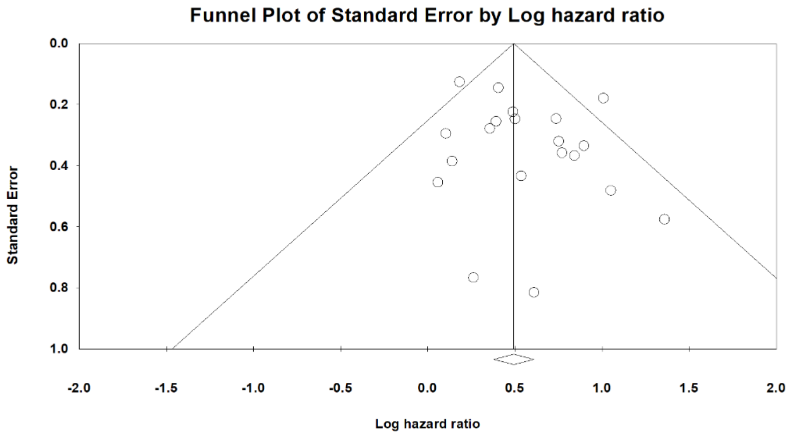

(C) HR of progression-free survival (Egger's test: p,0.10)

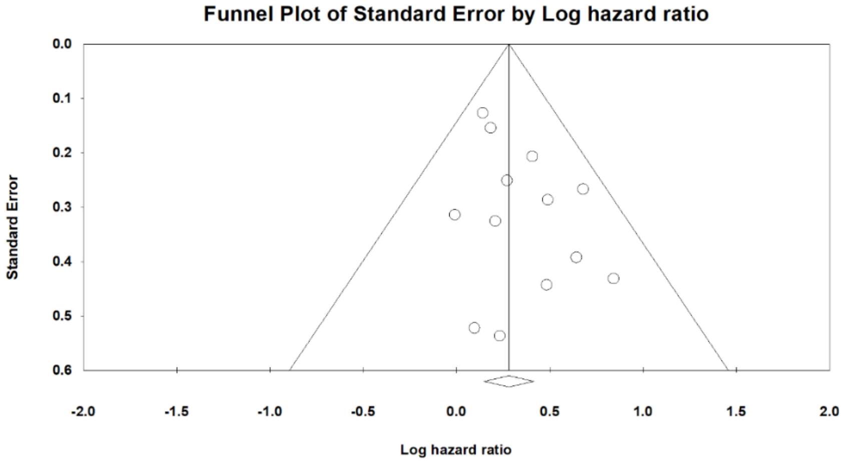

Figure S2. Sensitivity analysis

(A) Prevalence, (B) Overall survival, (C) Progression-free survival

(A) Prevalence

Event rate (95% CI) with study removed

Study name	Point	Lower limit	Upper limit
Hiraoka 2017	0.447	0.383	0.512
Naganumaa 2017	0.430	0.364	0.499
Nishikawa 2017	0.422	0.360	0.487
Antonelli 2018	0.431	0.364	0.501
Imai 2019	0.435	0.368	0.504
Labeur 2019	0.429	0.360	0.501
Sawada 2019	0.447	0.384	0.513
Wu 2021	0.452	0.392	0.514
Saeki 2021	0.430	0.360	0.504
Katsuaki 2022	0.424	0.359	0.492
Endo 2020	0.438	0.372	0.507
Hiraoka 2021	0.444	0.379	0.510
Dong 2022	0.428	0.362	0.496
Fujita 2022	0.431	0.363	0.502
Toshida 2022-a	0.424	0.359	0.492
Akce 2021	0.431	0.365	0.500
Kim 2021	0.446	0.382	0.512
Matsumoto 2022	0.434	0.368	0.502
Toshida 2022-b	0.428	0.363	0.496
Guo 2022	0.432	0.365	0.502
Chen 2023	0.430	0.363	0.500
Pooled	0.434	0.370	0.500

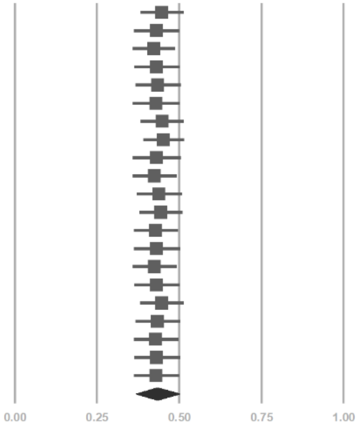

(B) overall survival

Hazard ratio (95% CI) with study removed

Study name	Point	Lower limit	Upper limit
Hiraoka 2017	1.68	1.44	1.97
Naganumaa 2017-M	1.68	1.44	1.96
Naganumaa 2017-F	1.70	1.46	1.98
Nishikawa 2017	1.54	1.37	1.74
Antonelli 2018	1.71	1.45	2.01
Imai 2019	1.67	1.43	1.94
Labeur 2019	1.78	1.56	2.04
Sawada 2019	1.72	1.48	2.01
Wu 2021	1.68	1.44	1.97
Saeki 2021	1.73	1.47	2.04
Katsuaki 2022	1.72	1.46	2.01
Endo 2020	1.72	1.48	2.00
Hiraoka 2021	1.71	1.45	2.00
Dong 2022	1.67	1.44	1.93
Fujita 2022	1.72	1.47	2.01
Toshida 2022-a	1.68	1.44	1.95
Akce 2021	1.70	1.46	1.99
Kim 2021	1.73	1.49	2.02
Toshida 2022-b	1.71	1.46	1.99
Chen 2023	1.68	1.43	1.96
Pooled	1.70	1.46	1.97

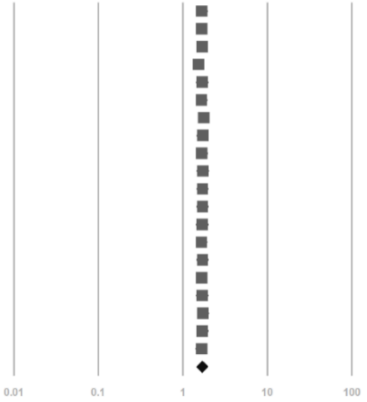

(C) Progression-free survival

Hazard ratio (95% CI) with study removed

Study name	Point	Lower limit	Upper limit
Nishikawa 2017	1.355	1.167	1.574
Sawada 2019	1.328	1.157	1.523
Wu 2021	1.307	1.138	1.500
Saeki 2021	1.402	1.195	1.644
Ogushi 2022	1.317	1.149	1.508
Dong 2022	1.304	1.138	1.494
Toshida 2022-a	1.308	1.142	1.499
Akce 2021	1.343	1.170	1.540
Kim 2021	1.324	1.152	1.523
Matsumoto 2022	1.328	1.159	1.520
Toshida 2022-b	1.324	1.157	1.516
Guo 2022	1.287	1.120	1.478
Chen 2023	1.303	1.130	1.502
Pooled	1.323	1.157	1.513

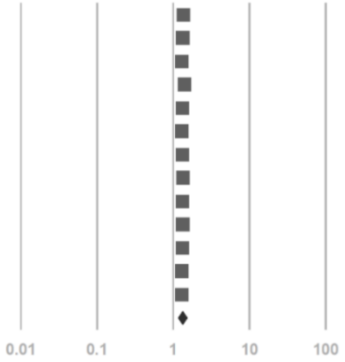

Supplement: Supplementary file 1 [file cancers-15-02426-s001.zip › cancers-2309593-supplementary.pdf]
